# Progress in symmetry preserving robot perception and control through geometry and learning

**DOI:** 10.3389/frobt.2022.969380

**Published:** 2022-09-14

**Authors:** Maani Ghaffari, Ray Zhang, Minghan Zhu, Chien Erh Lin, Tzu-Yuan Lin, Sangli Teng, Tingjun Li, Tianyi Liu, Jingwei Song

**Affiliations:** Computational Autonomy and Robotics Laboratory (CURLY), University of Michigan, Ann Arbor, MI, United States

**Keywords:** robot perception, robot control, geometric control, invariant extended Kalman filter, Lie groups, equivariant models, equivariant representation learning, deep learning

## Abstract

This article reports on recent progress in robot perception and control methods developed by taking the symmetry of the problem into account. Inspired by existing mathematical tools for studying the symmetry structures of geometric spaces, geometric sensor registration, state estimator, and control methods provide indispensable insights into the problem formulations and generalization of robotics algorithms to challenging unknown environments. When combined with computational methods for learning hard-to-measure quantities, symmetry-preserving methods unleash tremendous performance. The article supports this claim by showcasing experimental results of robot perception, state estimation, and control in real-world scenarios.

## 1 Introduction

Understanding the underlying principles of intelligence is at the heart of Artificial Intelligence (AI) and its applications for robotics, i.e., embodied AI, towards building a fully adaptive autonomous system capable of operating in the real world. Computational mathematics and intelligence have become a pivot for these fields, given the current advances in hardware. By combining, unifying, and expanding our mathematical and data-driven understanding of these areas of science and research, one can push the boundaries towards a unifying cognitive model that.

1. is robust to challenging environments and behavior modes;
2. takes into account hierarchical semantic knowledge of the scene such as objects and affordances as well as the geometry;
3. possesses sufficient mathematical and computational structures to be exploited for developing efficient and generalizable algorithms;
4. follows compositional principles to assemble integrated models that can produce outcomes bigger than the sum of individual modules.

This work provides an overview of our recent efforts for robot perception and control methods that can leverage structures such as symmetry and data simultaneously. Roughly speaking, symmetry of an object is a motion that leaves it unchanged (Tapp, 2021). For example, consider the sphere $S^{2}=\left\{\left(x_{1},x_{2},x_{3}\right)\in R^{3}|x_{1}^{2}+x_{2}^{2}+x_{3}^{2}=1\right\}$ . Its symmetry group is the three-dimensional orthogonal group O(3), i.e., the disjoint union of all 3D rotations and reflections. No matter how we rotate the sphere, its shape remains the same. More generally, Lie groups model the continuous symmetry of geometric spaces and are equipped with a natural coordinates system called exponential coordinates. An important consequence of this observation is that we can formulate problems more naturally where the Lie group action commutes with the (data-driven) functional representation of data (Section 2), the state estimation and control error dynamics become independent of the current operating point, and only depend on the desired relative motion (Sections 3 and Section 4), and we can lift multimodal signals, including images and point clouds, to some Lie algebras via equivariant networks (Section 5).

Section 2 presents a nonparametric analytical framework that models semantically labeled point clouds for solving the sensor registration problem (Ghaffari et al., 2019; Clark et al., 2021; Zhang et al., 2021). The framework lifts the data into a Reproducing Kernel Hilbert Space (RKHS), where the inner product structure captures the cross-correlation between two labeled point clouds as functions. This framework is an example of an equivariant model for modeling data where a Lie group transformation acts on these functions to align them.

Section 3 presents a robot state estimation framework using an invariant Kalman filtering (Barrau and Bonnabel, 2017; Barrau and Bonnabel, 2018; Hartley et al., 2020) and deep learning for estimating contact events from multi-modal proprioceptive sensory data (Lin et al., 2022). The novel combination of a geometric filter on Lie groups with deep learning to provide learned contact events without physical sensors show a promising direction on how to integrate real-time deep learning in high-frequency robot state estimation tasks.

Section 4 provides an overview of the error-state Model Predictive Control (MPC) on Lie groups and the stability analysis by a Lyapunov function expressed in the Lie algebra (Teng et al., 2022a; Teng et al., 2022b). We derive the linearized configuration error dynamics and equations of motion in the Lie algebra (tangent space at the identity) that, given an initial condition, are globally valid and independent of the system trajectory. This approach leads to a convex MPC algorithm for the tracking control problem using the linearized error dynamics, which can be solved efficiently using Quadratic Programming (QP) solvers. The proposed controller is validated in experiments on quadrupedal robot pose control and locomotion.

Section 5 presents recent frameworks for equivariant feature learning and their applications in registration and place recognition tasks (Zhu et al., 2022b). We learn an embedding for each input in a feature space that preserves the equivariance property, enabled by recent developments in symmetry-preserving neural networks. Symmetry (or equivariance) in a neural network enables efficient learning (by removing the need for data augmentation), generalization, and a clear connection between the changes in the input and output spaces, i.e., explainability.

Finally, Section 6 provides closing remarks by summarizing our new findings and their impacts on robot perception and control. We also discuss future opportunities enabled by the presented results in this article.

## 2 RKHS registration for spatial-semantic perception

Point clouds obtained by modern sensors such as RGB-D cameras, stereo cameras, and LIDARs contain up to 300, 000 points per scan at 10–60*Hz* and rich color and intensity (reflectivity of a material sensed by an active light beam) measurements besides the geometric information. In addition, *deep learning* (LeCun et al., 2015) can provide semantic attributes of the scene as measurements (Long et al., 2015; Chen et al., 2017; Zhu et al., 2019).

**FIGURE 1:**
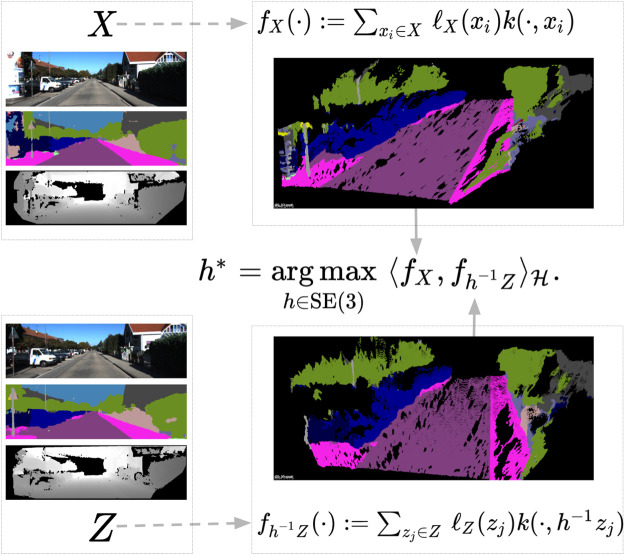
Point clouds *X* and *Z* are represented by two continuous functions *f*_*X*_, *f*_*Z*_ in an RKHS. Each point *x*_*i*_ has its own semantic labels, *ℓ*_*X*_(*x*_*i*_), encoded in the corresponding function representation via a tensor product representation (Zhang et al., 2021). The registration is formulated as maximizing the inner product between two point cloud functions.

Illustrated in Figure 1, the following formulation provides a general framework for lifting semantically labeled point clouds into a function space to solve a registration problem (Ghaffari et al., 2019; Clark et al., 2021; Zhang et al., 2021). Consider two (finite) collections of points, *X* = {*x*_*i*_}, $Z=\left\{z_{j}\right\}\subset R^{3}$ . We want to determine which element *h* ∈ SE(3), aligns the two point clouds *X* and *hZ* = {*hz*_*j*_} the “best.” To assist with this, we will assume that each point contains information described by a point in an inner product space, $\left(I,{\left\langle \cdot ,\cdot \right\rangle }_{I}\right)$ . To this end, we will introduce two labeling functions, $\ell_{X}:X\to I$ and $\ell_{Z}:Z\to I$ . To measure their alignment, we turn the point clouds, *X* and *Z*, into functions $f_{X},f_{Z}:R^{3}\to I$ that live in some RKHS, $\left(H,{\left\langle \cdot ,\cdot \right\rangle }_{H}\right)$ . The action, $\mathrm{SE}\left(3\right)↷R^{3}$ induces an action SE(3)↷H by *h*. *f*(*x*)≔*f*(*h* ^−1^ *x*). Inspired by this observation, we will set $h.f_{Z}≔f_{h^{-1}Z}$ .

Problem 1. *The problem of aligning the point clouds can now be rephrased as maximizing the scalar products of* *f*_*X*_ *and* *h*. *f*_*Z*_ *, i.e., we want to solve*

$$\underset{h\in \mathrm{SE}\left(3\right)}{\mathrm{arg max}}\;F\left(h\right),\;F\left(h\right)≔{\left\langle f_{X},f_{h^{-1}Z}\right\rangle }_{H}.$$ (1)

### 2.1 Constructing the functions

For the kernel of our RKHS, H , we first choose the squared exponential kernel $k:R^{3}\times R^{3}\to R$ :

$$k\left(x,z\right)=\sigma^{2}\exp\left(\frac{-\|x-z{\|}_{3}^{2}}{2\ell^{2}}\right),$$ (2)

for some fixed real parameters (hyperparameters) *σ* and *ℓ* (the *lengthscale*), and ‖ ⋅‖_3_ is the standard Euclidean norm on $R^{3}$ . This allows us to turn the point clouds to functions via $f_{X}\left(\cdot \right)≔\sum_{x_{i}\in X}\;\ell_{X}\left(x_{i}\right)k\left(\cdot ,x_{i}\right)$ and $f_{h^{-1}Z}\left(\cdot \right)≔\sum_{z_{j}\in Z}\;\ell_{Z}\left(z_{j}\right)k\left(\cdot ,h^{-1}z_{j}\right)$ . Here *ℓ*_*X*_(*x*_*i*_) encodes the semantic information, for example LIDAR intensity and image pixel color. *k*(⋅, *x*_*i*_) encodes the geometric information. We can now obtain the inner product of *f*_*X*_ and *f*_*Z*_ as

$$\langle f_{X},f_{h^{-1}Z}\rangle_{H}≔\underset{\begin{array}{c} x_{i}\in X,z_{j}\in Z \end{array}}{\sum }\langle \ell_{X}\left(x_{i}\right),\ell_{Z}\left(z_{j}\right)\rangle_{I}\cdot k\left(x_{i},h^{-1}z_{j}\right)$$ (3)

We use the kernel trick (Murphy, 2012) to substitute the inner products in (3) with the semantic kernel as ${\left\langle f_{X},f_{h^{-1}Z}\right\rangle }_{H}=\sum_{\begin{array}{c} x_{i}\in X,z_{j}\in Z \end{array}}k_{c}\left(\ell_{X}\left(x_{i}\right),\ell_{Z}\left(z_{j}\right)\right)\cdot k\left(x_{i},h^{-1}z_{j}\right)$ . We choose *k*_*c*_ to be the squared exponential kernel with real hyperparameters *σ*_*c*_ and *ℓ*_*c*_ that are set independently.

### 2.2 Feature embedding via tensor product representation

We now extend the feature space to a hierarchical distributed representation to incorporate the full geometric and hierarchical semantic relationship between the two point clouds. Let (*V*_1_, *V*_2_, … ) be different inner product spaces describing different types of non geometric features of a point, such as color, intensity, and semantics. Their tensor product, *V*_1_ ⊗ *V*_2_ ⊗… is also an inner product space. For any *x* ∈ *X*, *z* ∈ *Z* with features *ℓ*_*X*_(*x*) = (*u*_1_, *u*_2_, … ) and *ℓ*_*Z*_(*z*) = (*v*_1_, *v*_2_, … ), with *u*_1_, *v*_1_ ∈ *V*_1_, *u*_2_, *v*_2_ ∈ *V*_2_, … , we have

$$\langle \ell_{X}\left(x\right),\ell_{Z}{\left(z\right)\rangle }_{I}=\langle u_{1}⊗u_{2}⊗\ldots ,v_{1}⊗v_{2}⊗\ldots \rangle =\langle u_{1},v_{1}\rangle \cdot \langle u_{2},v_{2}\rangle \cdot \ldots .$$ (4)

By substituting (4) into (3), we obtain ${\left\langle f_{X},f_{h^{-1}Z}\right\rangle }_{H}=\sum_{\begin{array}{c} x_{i}\in X,z_{j}\in Z \end{array}}\left\langle u_{1i},v_{1j}\right\rangle \cdot \left\langle u_{2i},v_{2j}\right\rangle \ldots k\left(x_{i},h^{-1}z_{j}\right)$ . After applying the kernel trick we arrive at

$${\left\langle f_{X},f_{h^{-1}Z}\right\rangle }_{H}=\underset{\begin{array}{c} x_{i}\in X,z_{j}\in Z \end{array}}{\sum }\;k\left(x_{i},h^{-1}z_{j}\right)\cdot \underset{k}{\prod }k_{V_{k}}\left(u_{ki},v_{kj}\right)≔\underset{\begin{array}{c} x_{i}\in X,z_{j}\in Z \end{array}}{\sum }\;k\left(x_{i},h^{-1}z_{j}\right)\cdot c_{ij}.$$ (5)

Each *c*_*ij*_ does not depend on the relative transformation. It is worth noting that, when choosing the squared exponential kernel and when the input point clouds have only geometric information, *c*_*ij*_ will be identity, and (5) has the same formulation as Kernel Correlation (Tsin and Kanade, 2004).

### 2.3 Equivariance property

If instead of working with the inverse of the transformation acting on the function basis we work with the function input, then the equivariance property becomes evident. Let $C\left(R^{3}\right)$ be the set of point clouds on $R^{3}$ and H be the RKHS. Let $f:C\left(R^{3}\right)\to H$ be our map which assigns a function to a point cloud. Consider the space of smooth functions on $R^{3}$ , $C^{\infty }\left(R^{3}\right)$ , and let the group G act on $R^{3}$ . The action lifts to an action on $C^{\infty }\left(R^{3}\right)$ via *g*. *f*(*x*) = *f*(*g* ^−1^ *x*), g∈G . This inverse is needed to make the action a *group* action:

$$\left(hg\right).f\left(x\right)=h.f\left(g^{-1}x\right)=f\left(g^{-1}h^{-1}x\right)=f\left({\left(hg\right)}^{-1}x\right),\;h,g\in G.$$

Now let *Z* be a point cloud and *f*_*Z*_ be its associated function. If G acts on $R^{3}$ via isometries, then *k*(*gx*, *gz*) = *k*(*x*, *z*) and we have

$$g.f_{Z}\left(x\right)=f_{Z}\left(g^{-1}x\right)=\underset{j}{\sum }\;\ell_{Z}\left(z_{j}\right)\cdot k\left(g^{-1}x,z_{j}\right)=\underset{j}{\sum }\;\ell_{j}\cdot k\left(x,gz_{j}\right)=f_{gZ}\left(x\right).$$

### 2.4 Experimental results

We present the point cloud registration experiments on real world outdoor and indoor datasets: KITTI (Geiger et al., 2012) odometry and TUM RGB-D data set (Sturm et al., 2012), with the following setup: All experiments are performed in a frame-to-frame manner without skipping images. The first frame’s transformation is initialized with identity, and all later frames start with the previous frames’ results. The same hyperparameter values such as lengthscale of the kernels in (2) are used for the proposed registration methods within one data set. All the baselines except Robust-ICP (Zhang et al., 2022) use all the pixels without downsampling because they do not provide an optimal point selection scheme. Fast-Robust-ICP and the proposed methods select a subset of pixels via OpenCV’s FAST (Rosten and Drummond, 2006) feature detector to reduce the frame-wise running time.

**FIGURE 2:**
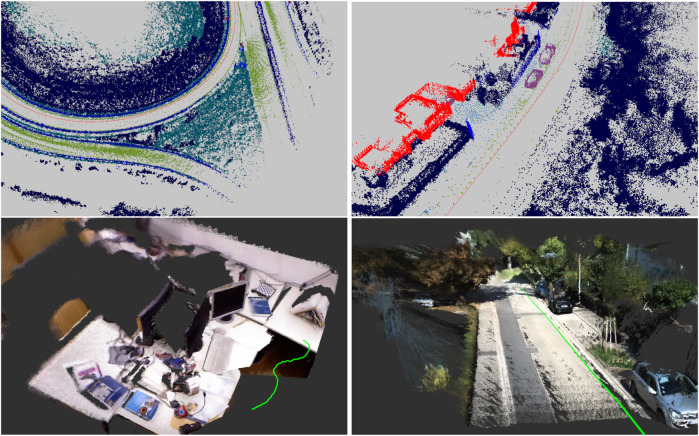
Stacked semantic and color point clouds based on frame-to-frame registration results using KITTI (Geiger et al., 2012) LiDAR, TUM RGB-D (Sturm et al., 2012) and KITTI Stereo sensors.

**FIGURE 3:**
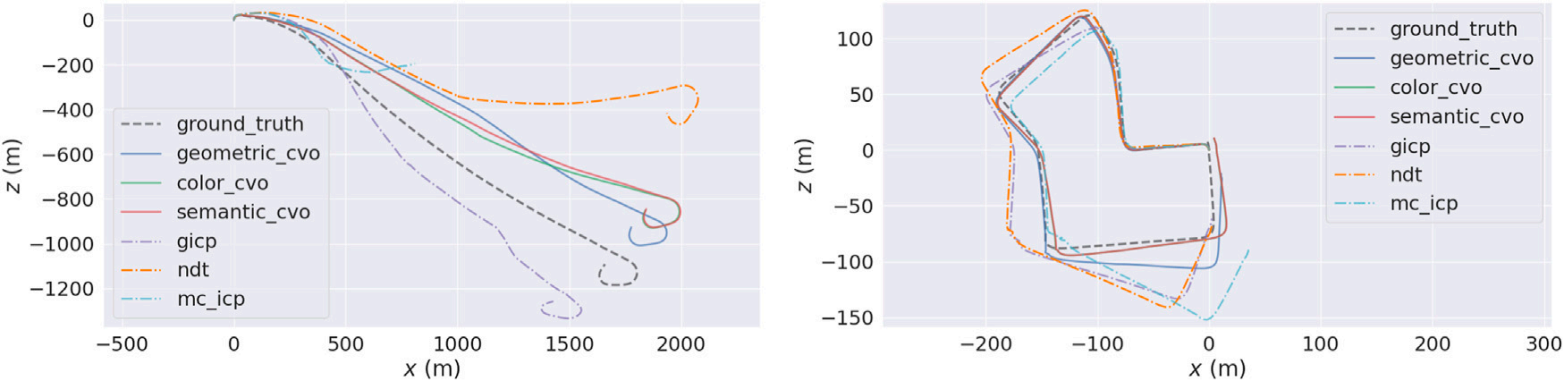
An illustration of the proposed registration methods on KITTI Stereo (Geiger et al., 2012) sequence 01 (left) and 07 (right) versus the baselines. The black dashed trajectory is the ground truth. The dot-dashed trajectories are the baselines. Plotted with EVO (Grupp, 2017).

The qualitative and quantitative results on KITTI Stereo is provided in Figure 2, Figure 3, and Table 1, respectively. The baselines are GICP (Segal et al., 2009), Multichannel-ICP (Servos and Waslander, 2014), 3D-NDT (Magnusson et al., 2007), and Robust-ICP (Zhang et al., 2022). GICP and NDT are compared with our geometric registration method (*Geometric CVO*, i.e., *ℓ*_*X*_(*x*_*i*_) = *ℓ*_*Z*_(*z*_*j*_) = 1). Multichannel-ICP competes with our color-assisted registration method (*Color CVO*). GICP and 3D-NDT implementation are from PCL (Rusu and Cousins, 2011). The Robust-ICP implementation is from its open source Github repositiory. The Multichannel-ICP implementation is from (Parkison et al., 2019). The semantic predictions of the images come from Nvidia’s pre-trained neural network (Zhu et al., 2019), which was trained on 200 labeled images on KITTI. The depth values of the stereo images are generated with ELAS (Geiger et al., 2010). All the baselines and the proposed methods remove the first 100 rows of image pixels that mainly include sky pixels, as well as points that are more than 55 m away. Averaged over sequence 00 to 10, our geometric method has a lower translational error (4.55%) comparing to the GICP (11.23%), NDT (8.50%), and Robust-ICP (11.02%). Our color version has a lower average translational drift (3.69%) than Multichannel-ICP (14.10%). If we add semantic information the error is further reduced (3.64%). In addition, excluding the image I/O and point cloud generation operations, the proposed implementations takes on average 1.4 s per frame on GPU when registering less than 15k downsampled points. Fast-Robust-ICP also takes downsampled point clouds and takes 0.3 s per frame on CPU. GICP, NDT, and Multichannel-ICP on CPU use full point clouds (150k-350k points), and take 6.3, 6.6, and 57 s per frame, respectively.

**TABLE 1:** Results of the proposed frame-to-frame method using the KITTI (Geiger et al., 2012) stereo odometry benchmark averaged over Sequence 00–10. The table lists the average drift in translation, as a percentage (%), and rotation, in degrees per meter(°/*m*). The drifts are calculated for all possible subsequences of 100, 200 …., 800 m.

	**t** (%)	**r** (°/*m*)
Geometric Registration (Proposed)	4.55	0.0236
GICP Segal et al. (2009)	11.23	0.0452
3D-NDT Magnusson et al. (2007)	8.50	0.0396
Robust-ICP Zhang et al. (2022)	11.02	0.0256
Color Registration (Proposed)	3.69	0.0159
MC-ICP Servos and Waslander, (2014)	14.10	0.0488
Semantic Registration (Proposed)	**3.64**	**0.0155**

The qualitative and quantitative results on TUM RGB-D is provided in Figure 2 and Table 2, respectively. We evaluated our method on the fr1 sequences, which are recorded in an office environment, and fr3 sequences, which contain image sequences in structured/nostructured and texture/notextured environments. We use the same baselines for geometric registration as KITTI. We compare Color CVO with Dense Visual Odometry (DVO) (Kerl et al., 2013) and Color ICP (Park et al., 2017). We reproduced DVO results with the code from (Pizenberg, 2019). The Color ICP implementation is taken from Open3D (Zhou et al., 2018). From Table 2, the proposed geometric registration outperforms the geometric baselines and achieves a similar performance to DVO and Color ICP. Moreover, with color information, the average error of the proposed registration decreases.

**TABLE 2:** The RMSE of Relative Pose Error (RPE) averaged over TUM RGB-D (Sturm et al., 2012) fr1 and fr3 structure v.s texture sequences. The **t** columns show the RMSE of the translational drift in m/sec and the **r** columns show the RMSE of the rotational error in deg  /sec. The RMSE is averaged over all sequences.

	fr1		fr3 structure v.s texture	
	t (m/sec)	r (deg /sec)	t (m/sec)	r (deg /sec)
Geometric Registration (Proposed)	0.0730	**2.3805**	0.0794	2.8536
GICP Segal et al. (2009)	0.4034	15.8838	0.2116	5.2979
3D-NDT Magnusson et al. (2007)	0.2290	14.0311	0.2487	6.9860
Robust-ICP Zhang et al. (2022)	0.1487	6.6911	0.2091	5.4168
Color Registration (Proposed)	**0.0545**	2.4333	**0.0754**	2.6651
DVO Kerl et al. (2013)	0.0623	2.6943	0.1386	4.9843
Color ICP Park et al. (2017)	0.1353	5.8985	0.0820	**2.2041**

### 2.5 Discussions and limitations

Results in Section 2.4 demonstrate that embedding features like color and semantics in function representations provide finer data associations. Specifically, in (5), the extra appearance information *c*_*ij*_ encodes the similarity in color or semantics between the two associated points. It eliminates pairwise associations whose color or semantic appearances do not agree. Moreover, each point *x*_*i*_ ∈ *X* is matched to multiple points *z*_*j*_ ∈ *Z*. The proposed color registration significantly improves over geometric-only methods in both KITTI Stereo and TUM RGB-D datasets.

One limitation of the proposed method is the computational complexity introduced by the double sum in (5). However, the double sum is sparse because a point *x*_*i*_ ∈ *X* is far away from the majority of the points *z*_*j*_ ∈ *Z*, either in the spatial (geometry) space or one of the feature (semantic) spaces. But this similarity still has to be calculated with the help of GPU implementations or K-nearest-neighbor search (Blanco and Rai, 2014). In practice, an efficient point selection mechanism like FAST (Rosten and Drummond, 2006) corner selector or DSO’s (Engel et al., 2017) image gradient-based pixel selector can reduce the computation time. Alternatively, representation learning can be a way to reduce the number of input points while providing richer features.

## 3 Learning-aided invariant robot sate estimation

Matrix Lie groups (Chirikjian, 2011; Hall, 2015; Barfoot, 2017) provide natural (exponential) coordinates that exploits symmetries of the space (Long et al., 2013; Barfoot and Furgale, 2014; Forster et al., 2016; Mangelson et al., 2020; Mahony and Trumpf, 2021; Brossard et al., 2022). State estimation is the problem of determining a robot’s position, orientation, and velocity that are vital for robot control (Barfoot, 2017). An interesting class of state estimators that can be run at high frequency, e.g., 2 kHz, are based on Invariant Extended Kalman Filter (InEKF) (Barrau, 2015; Barrau and Bonnabel, 2017; Barrau and Bonnabel, 2018). The theory of invariant observer design is based on the estimation error being invariant under the action of a matrix Lie group. The fundamental result is that by correct parametrization of the error variable, a wide range of nonlinear problems can lead to (log) linear error dynamics (Bonnabel et al., 2009; Barrau, 2015; Barrau and Bonnabel, 2017).

Proprioceptive state estimators often combine data from an Inertial Measurement Unit (IMU) with signals such as body velocity, kinematics information, and contact events. A successful method in this domain for legged robots is the contact-aided InEKF (Hartley et al., 2020). This approach is attractive because the odometry estimate only depends on inertial, contact, and kinematic data, which barring sensor failure, always exist. Furthermore, the independence from any vision systems make the state estimator robust to perceptually degraded situations (Hartley et al., 2018; Lin et al., 2022). Many existing perception and navigation methods can work well, given a correct though uncertain initial condition; hence, such an accurate dead reckoning can enable higher levels of autonomy for existing systems.

The invariant observer design provides us with a framework with better convergence properties. However, sensory data input likewise plays a crucial role in state estimation tasks. Noisy and biased measurements can hinder the performance of the observer. On the other hand, sensor failures can lead to catastrophic results in state estimation. Recent deep learning methods allow one to address these challenges by estimating the bias or inferring the information that traditional sensors cannot obtain (Liu et al., 2018; Wellhausen et al., 2019). By combining learning with the symmetry-preserving observer design, the performance and robustness of a state estimator can be greatly improved (Brossard et al., 2019; Brossard et al., 2020).

This section reports our recent developments on deep-learning-aided invariant state estimator (Lin et al., 2022). In this work, a deep contact estimator is designed to estimate the foot contact events for legged robots. The learned foot contacts are then used to enforce the non-slip constraint in an InEKF. Although the complete state estimation pipeline is purely proprioceptive, it can achieve a similar performance to a state-of-the-art visual SLAM system. In addition, the program, including the deep contact estimator, runs in real-time (500 Hz) on an MIT Mini Cheetah robot. We also report our new results on developing the InEKF for wheeled platforms in Section 3.4. The data sets and software are available for download .

### 3.1 Deep contact estimator

The goal of the deep contact estimator is to accurately estimate the foot contact events where the robot’s foot maintain zero velocity in the world frame. We model the contact as binary events on each leg *l* ∈ {*RF*, *LF*, *RH*, *LH*}. The overall contact states of the robot becomes a collection of binary values $C=\left[\begin{array}{cccc} c_{RF} & c_{LF} & c_{RH} & c_{LH} \end{array}\right]$ , where *c*_*l*_ ∈ {0, 1} with 0 indicates no contact, and 1 denotes a firm contact. For a quadruped robot, there exist 16 different combinations of the contact states. We formulate our approach as a classification task^1^.

**FIGURE 4:**
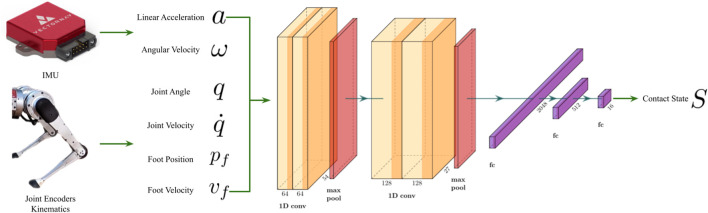
The architecture of the proposed contact estimator (Lin et al., 2022). The inputs include linear accelerations and angular velocities from an IMU, joint angles and joint velocities from encoders, and foot positions and velocities from kinematics.

The contact estimator takes sensor measurements from an IMU, joint encoders, and kinematics as input. To allow the network to extract information from the time domain, a fixed number of past data is concatenated together before inputting into the network. Figure 4 lists the input data along with the network architecture. The linear block contains 3 fully-connected layers that convert the deep features into the 16 classes. Dropout mechanisms are also added to the first 2 fully-connected layers to prevent the network from overfitting. Finally, we employ the cross-entropy loss for the classification task.

### 3.2 Contact data sets

**FIGURE 5:**
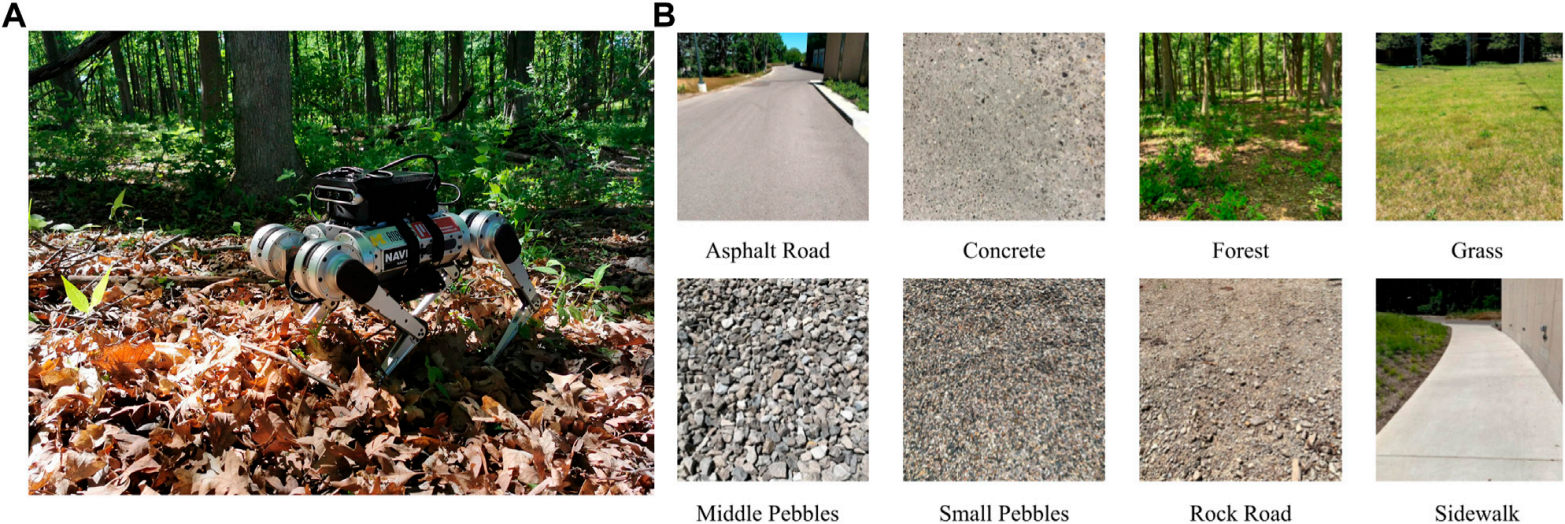
**(A)** Setup of an MIT Mini Cheetah with the perception suite used in the data collection. **(B)** Different ground types in the contact data set.

We create open-sourced contact data sets using an MIT Mini Cheetah robot (Katz et al., 2019). The data sets are collected using an MIT controller (Kim et al., 2019) across 8 different terrains (shown in Figure 5). We record proprioceptive measurements such as joint encoders data, foot positions and velocities, IMU measurements, and estimated joint torques from the controller. The IMU measurements are received at 1000*Hz*, while other data are recorded at 500*Hz*. We upsample all measurements to match the IMU frequency after recording the data. In addition to the proprioceptive measurements, we also record RGB-D images with an Intel D455 camera mounted on top of the robot. These RGB-D images are used in a state-of-the-art visual SLAM algorithm, ORB SLAM2 (Mur-Artal and Tardós, 2017). For the grass data sets, we obtain ground truth trajectories from a motion capture system. However, for the rest of the data sets, we use the trajectory from ORB SLAM2 as an approximation to ground truth. In total, around 1,000,000 data points were collected on 8 different terrains. We also include some examples of the robot walking in the air to provide the network with negative examples by holding the robot up and applying the same controller commands. The detailed number of data collection is listed in Table 3. The labels of the ground truth contacts are generated automatically with an offline pre-processing algorithm (self-supervised learning). Detailed of the algorithm can be found in the work of (Lin et al., 2022).

**TABLE 3:** Number of data of each terrain in the contact data sets.

Terrain Type										
overall	air trotting	air pronking	asphalt road	concrete	forest	grass	middle pebble	small pebble	rock road	sidewalk
1,013,441	44,386	48,972	94,615	465,144	72,144	103,392	44,442	52,669	45,819	58,115

### 3.3 Experimental results

We evaluate the accuracy, false positive rate, and false negative rate of the proposed contact estimator using the Mini Cheetah robot, as shown in Table 4. We compare our method with a model-based approach (Focchi et al., 2013; Fakoorian et al., 2016; Fink and Semini, 2020), denoted GRF Thresholding, and a fixed gait cycle assumption which assume the pre-determined gait cycle is precisely followed by the controller. Our method performs the best across all three sequences. It is worth noticing that the proposed contact estimator has the lowest false positive rate, which is crucial for state estimation tasks as the violation of the non-slip condition could lead to severe drift in the estimation.

**TABLE 4:** Accuracy comparison against baselines. The proposed method achieves the highest accuracy on all sequences. Although the gait cycle method has an accuracy closer to the proposed method, it does not remove false positives when gait cycle is violated.

Sequence	Method	% Accuracy					% False Positive Rate	% False Negative Rate
		Leg RF	Leg LF	Leg RH	Leg LH	Leg Avg	Leg Avg	Leg Avg
Concrete Short Loop	GRF Thresholding	73.43	70.02	71.69	70.04	71.30	37.07	13.24
	Gait Cycle	85.66	84.98	84.68	85.11	85.11	22.95	**0.00**
	Proposed Method	**98.34**	**97.87**	**97.95**	**98.56**	**98.18**	**1.45**	2.51
Grass Test Sequence	GRF Thresholding	82.55	78.93	84.62	82.48	82.14	26.87	**0.63**
	Gait Cycle	92.41	92.38	91.04	90.55	91.59	10.95	3.53
	Proposed Method	**98.08**	**97.57**	**97.73**	**97.73**	**97.78**	**2.35**	1.98
Forest Test Sequence	GRF Thresholding	80.99	80.09	82.75	83.24	81.77	26.54	1.84
	Gait Cycle	83.03	82.56	84.44	84.28	83.58	24.71	**0.08**
	Proposed Method	**97.05**	**96.62**	**97.24**	**97.40**	**97.08**	**2.82**	3.12

Bold values in tables show the best performance.

**FIGURE 6:**
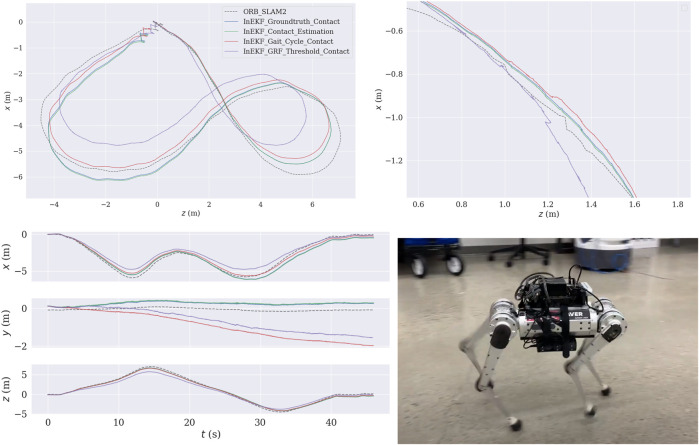
Concrete short loop test sequence. Top Left: The bird’s-eye view of the trajectories. The estimated trajectory is mapped to the camera frame (*Y* pointing downward, and *Z* pointing forward). Top Right: Zoomed-in of the bird’s-eye view. Bottom Left: This figure shows that the gait cycle and GRF thresholding methods produce a significant height (Y) drift. Bottom Right: Robot configuration.

We integrated our contact estimator into the contact-aided InEKF. The entire state estimation pipeline, including our deep contact estimator, runs in real-time at 500 *Hz* on an NVIDIA Jetson AGX Xavier. Figure 6 shows the trajectory generated by the InEKF using different contact sources on a concrete loop sequence. We also run the filter using the ground truth contact data to serve as a reference. Qualitatively compared to the baseline contact detectors, the resulting trajectory with the proposed contact estimation has smaller drifts from the trajectory with ground truth contacts, especially in the height (Y) axis. Furthermore, compared to the baseline contact estimators, the proposed method also yields a smoother trajectory.

### 3.4 Invariant EKF with body velocity measurements

**FIGURE 7:**
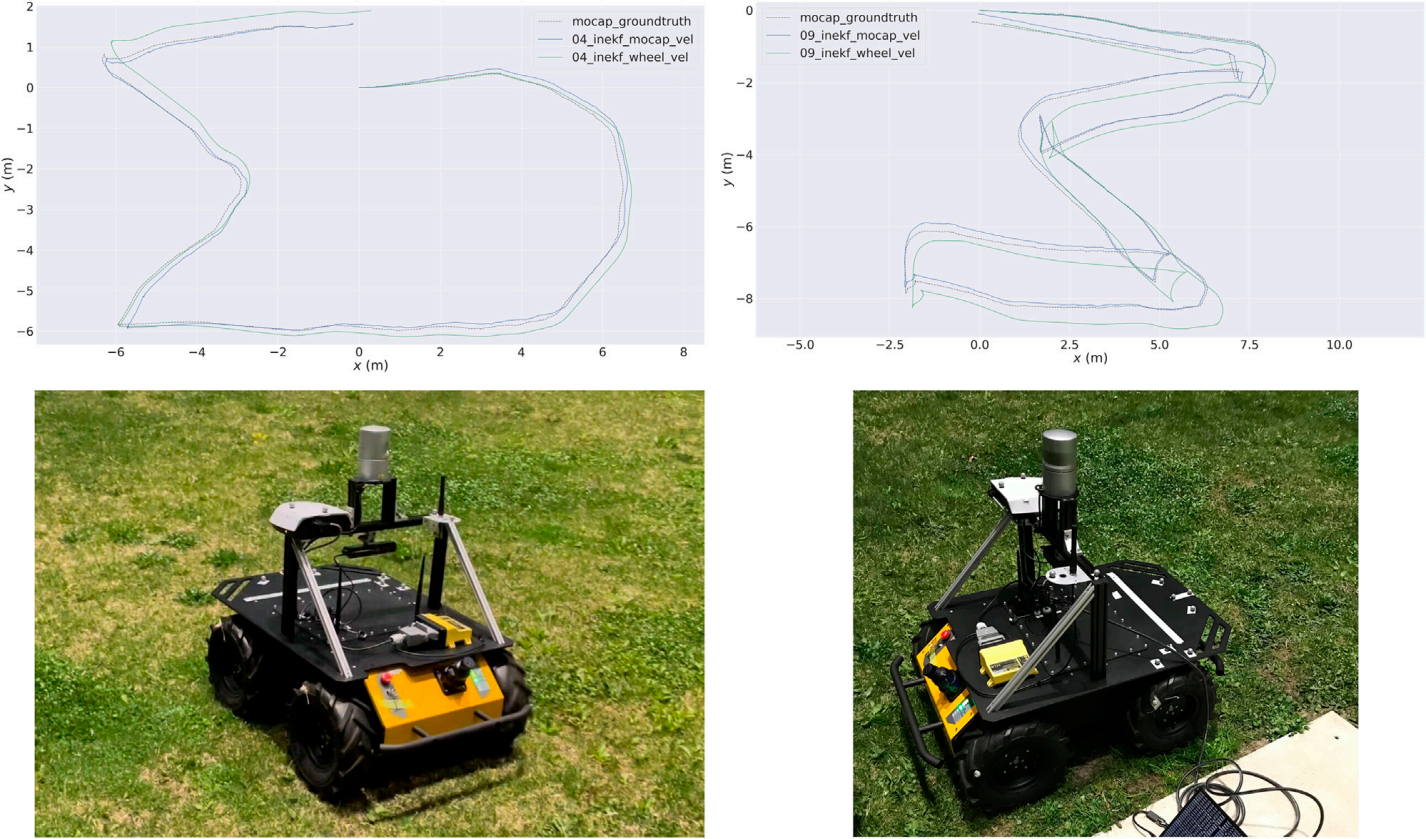
Top: Two sequences of trajectories recorded at the University of Michigan MAir motion capture facility. The green lines are the InEKF estimated trajectories using velocity estimated from the wheel encoders, and the blue lines are the InEKF results using velocity from the motion capture system. Bottom: The robot setup. The ground of the facility is planted with natural grass.

In addition to legged robots, we also develop state estimation software for wheeled robots using the InEKF. Instead of using the foot contact, here we use the body velocity as measurements in the correction step. Although the implementation is not restricted to a specific platform, we evaluate the performance of the filter on a differential-drive wheeled robot, Husky, from Clearpath robotics. We obtain the body velocity measurements from wheel encoders using a simple differential-drive model, $v_{\text{body}}=\frac{r\left(\omega_{l}+\omega_{r}\right)}{2}$ , where *ω*_*l*_ and *ω*_*r*_ are wheel angular velocities measured by the wheel encoders and *r* is the wheel radius. Moreover, we also use pseudo velocity measurements by assuming zero velocities on the *Y* and *Z* axis (Dissanayake et al., 2001). However, this estimation can be noisy and inaccurate due to slip or bumping on the wheels. In order to know the full potential of this framework, we also record several sequences in a motion capture facility and use the velocity from the motion capture system to correct the estimated state. Figure 7 shows the resulting trajectories. Using the wheel velocity and pseudo velocity measurements, the state estimator can produce a good estimation of the robot pose. If the accuracy of the velocity is improved, then the drift can be further reduced.

Although this section does not discuss the incorporation of learning into the InEKF state estimator, as done previously for the legged robot, the following lessons from our experiments are noteworthy.

• Body velocity measurements provide a generic correction model that can work on any robotic platform. However, accurate body velocity measurement is not readily available. Specifically, the filer requires the ground referenced body velocity (Teng et al., 2021b; Potokar et al., 2021).
• The robot’s nonholonomic constraints (i.e., velocity constraints that cannot be integrated) can provide pseudo observations that can significantly improve the performance. However, these constraints are assumptions and detached from the robot’s behavior. Learning such constraints provides a way to use sensory inputs instead of assumptions (Brossard et al., 2019; Brossard et al., 2020).
• Moreover, the nonholonomic constraints are violated when the robot drifts. Slip detection and friction estimation are challenging and necessary tasks for future learning-aided robot estimation modules.

## 4 Symmetry-preserving geometric robot control

The geometry of the configuration space of a robotics system can naturally be modeled using matrix Lie (continuous) groups (Bloch, 2015; Lynch and Park, 2017). For example, the centroidal dynamics of legged robots can be approximated by a single rigid body, whose motion is on SE(3).

The Euler angle based convex Model Predictive Control (MPC) (Di Carlo et al., 2018) has been proposed for locomotion planning on the quadrupedal robot. Zero roll and pitch angle assumptions are validated by assuming a flat ground, which may fail when such assumptions no longer hold. To avoid the problem, the geometric MPC that utilize the symmetry of the Lie group has been proposed. A local control law has been proposed by Kalabić et al. (2016); Kalabić et al. (2017), where the linearized dynamics are defined by a local diffeomorphism from the SE(3) manifold to $R^{n}$ space. However, such a diffeomorphism is not unique and too abstract for controller design.

**FIGURE 8:**
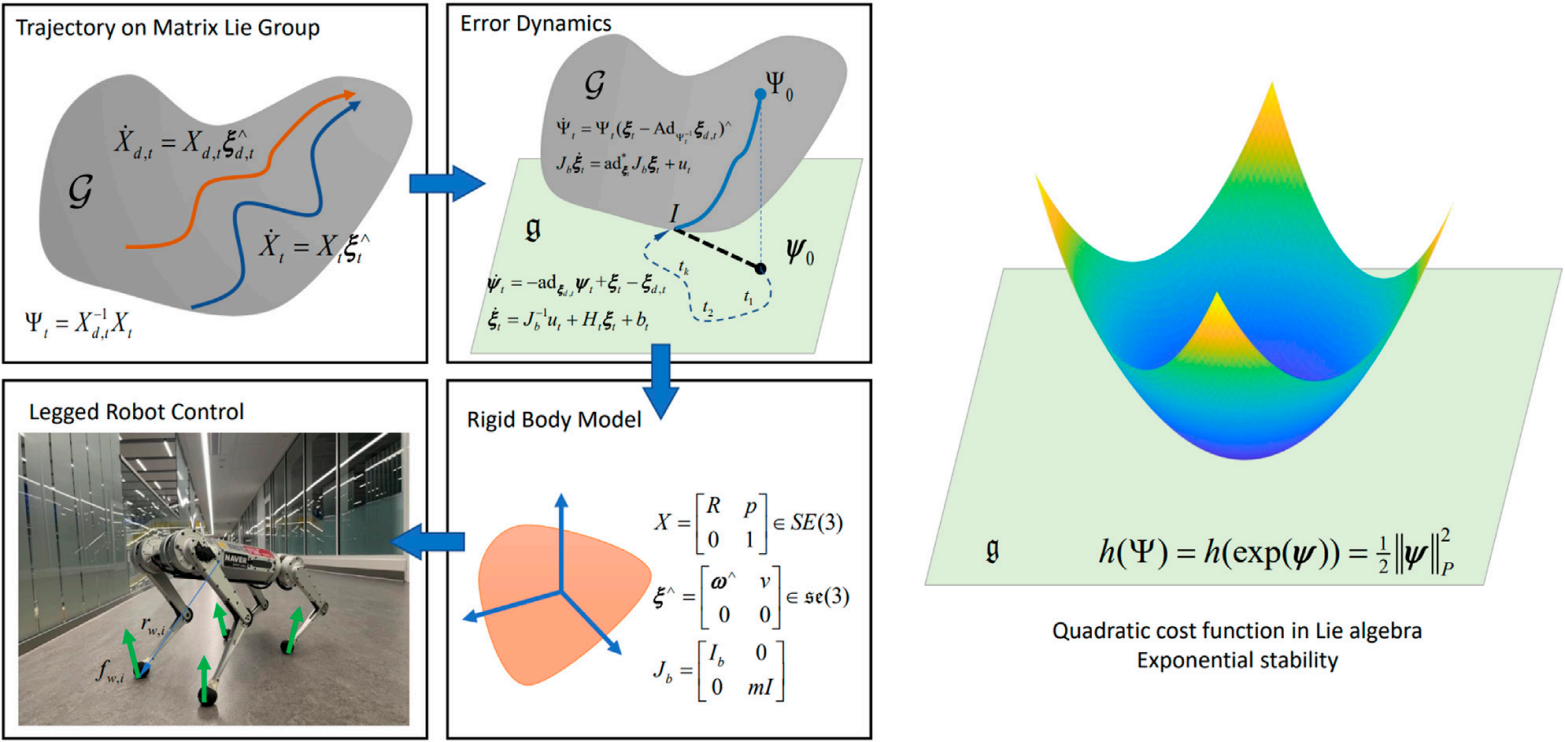
The proposed error-state MPC framework by Teng et al. (2022a). The tracking error is defined on a matrix Lie group and linearized in the Lie algebra. A convex MPC algorithm is derived via the linearized dynamics for tracking control. The proposed algorithm is applied to a single rigid body system and verified on a quadrupedal robot MIT Mini Cheetah. A quadratic cost function in the Lie algebra can verify the stability of the proposed MPC.

The Variational Based Linearization (VBL) technique (Wu and Sreenath, 2015) are applied to linearize the Lagrangian to obtain the discrete-time equation of motion and applied to robot pose control (Chignoli and Wensing, 2020). A VBL based MPC is proposed by Agrawal et al. (2021) for locomotion on discrete terrain using a gait library. The result suggests that the VBL based linearization can preserve the energy, thus making the system more stable. However, the VBL method linearized the system at the reference trajectory, which may result in unstable motion (Ding et al., 2021). Other than linearizing at the reference trajectory, the work of Ding et al. (2021) linearized the system at the current operating point to obtain the QP problem for tracking of legged robot trajectory. However, the linearized state matrix of Ding et al. (2021) depends on the orientation, which can be avoided by exploiting the symmetry of the system as done by Teng et al. (2022a,b). The proposed framework is illustrated in Figure 8.

### 4.1 Error-state convex MPC

For tracking control on Lie group G , we define the desired trajectory as $X_{d,t}\in G$ and the actual state as $X_{t}\in G$ , both as function of time *t*. Given the twists *ξ*_*t*_ and desired twists *ξ*_*d*,*t*_ and the reconstruction equation, we have $\frac{d}{dt}X_{t}=X_{t}\xi_{t}^{\wedge },\;\frac{d}{dt}X_{d,t}=X_{d,t}\xi_{d,t}^{\wedge }$ . Similar to the left or right error defined in (Bullo and Murray, 1999), we define the error between $X_{t}^{d}$ and *X*_*t*_ as

$$\Psi_{t}=X_{d,t}^{-1}X_{t}\in G.$$ (6)

For the tracking problem, our goal is to drive the error from the initial condition Ψ_0_ to the identity I∈G . Taking derivative on both sides of (6), we have

$$\begin{array}{ll} \frac{d}{dt}\Psi_{t} & ={\dot{\Psi }}_{t}=\frac{d}{dt}\left(X_{d,t}^{-1}\right)X_{t}+X_{d,t}^{-1}\frac{d}{dt}X_{t}=X_{d,t}^{-1}\frac{d}{dt}X_{t}-X_{d,t}^{-1}\frac{d}{dt}\left(X_{d,t}\right)X_{d,t}^{-1}X_{t}\\ & =X_{d,t}^{-1}X_{t}\xi_{t}^{\wedge }-X_{d,t}^{-1}X_{d,t}\xi_{d,t}^{\wedge }X_{d,t}^{-1}X_{t}=\Psi_{t}\xi_{t}^{\wedge }-\xi_{d,t}^{\wedge }\Psi_{t}.\\ {\dot{\Psi }}_{t} & =\Psi_{t}{\left(\xi_{t}-\Psi_{t}^{-1}\xi_{d,t}\Psi_{t}\right)}^{\wedge }=\Psi_{t}{\left(\xi_{t}-{Ad}_{\Psi_{t}^{-1}}\xi_{d,t}\right)}^{\wedge }. \end{array}$$ (7)

We define $\psi_{t}^{\wedge }$ as an element of the Lie Algebra that corresponds to Ψ_*t*_. Thus by the exponential map, we have $\Psi_{t}=\exp\left(\psi_{t}\right),\;\Psi_{t}\in G,\;\psi_{t}^{\wedge }\in g$ . Given the first-order approximation of the exponential map, $\Psi_{t}=\exp\left(\psi_{t}\right)\approx I+\psi_{t}^{\wedge },$ and a first-order approximation of the adjoint map ${Ad}_{\Psi_{t}}\approx {Ad}_{I+{\psi_{t}}^{\wedge }}$ , we can linearize (7) by only keeping the first order term of *ψ*_*t*_ and *ξ*_*t*_ − *ξ*_*d*,*t*_ as:

$${\dot{\Psi }}_{t}\approx \left(I+{\dot{\psi }}_{t}^{\wedge }\right)\approx \left(I+\psi_{t}^{\wedge }\right){\left(\xi_{t}-{Ad}_{\left(I-\psi_{t}^{\wedge }\right)}\xi_{d,t}\right)}^{\wedge },$$ (8)

$${\dot{\psi }}_{t}=-{ad}_{\xi_{d,t}}\psi_{t}+\xi_{t}-\xi_{d,t}.$$ (9)

Eq. 9 is the linearized velocity error in the Lie algebra.

The dynamics of *ξ*_*t*_ is described by the *forced* Euler-Poincaré equations (Bloch et al., 1996; Bloch, 2015) as $J_{b}\dot{\xi }={ad}_{\xi }^{*}J_{b}\xi +u$ , where $u\in g^{*}$ is the generalized control input force applied to the body fixed principal axes, ad∗ is the co-adjoint action, and $g^{*}$ is the cotangent space. This model is nonlinear. To compute a locally linear approximation of the nonlinear term, we adopt the Jacobian linearization around the operating point $\bar{\xi }$ as $J_{b}\dot{\xi }\approx {ad}_{\bar{\xi }}^{*}J_{b}\bar{\xi }+\frac{\partial {ad}_{\xi }^{*}J_{b}\xi }{\partial \xi }{|}_{\bar{\xi }}\left(\xi -\bar{\xi }\right)+u$ . Thus, we have the linearized dynamics in the following form $\dot{\xi }=H_{t}\xi +J_{b}^{-1}u+b_{t}$ , We define the system states as $x_{t}≔\left[\begin{array}{c} \psi_{t}\\ \xi_{t} \end{array}\right]$ . Then, the linearized dynamics becomes ${\dot{x}}_{t}=A_{t}x_{t}+B_{t}u_{t}+h_{t}$ , where

$$A_{t}≔\left[\begin{array}{cc} -{ad}_{\xi_{d,t}} & I\\ 0 & H_{t} \end{array}\right],B_{t}≔\left[\begin{array}{c} 0\\ J_{b}^{-1} \end{array}\right],h_{t}≔\left[\begin{array}{c} -\xi_{d,t},\\ b_{t} \end{array}\right].$$

### 4.2 Convex MPC design

On Lie groups, our cost function is designed to regulate the tracking error *ψ*_*t*_ and its derivative ${\dot{\psi }}_{t}$ rather than the difference between *ξ*_*d*,*t*_ and *ξ*_*t*_. Thus, our tracking error can be designed as:

$$y_{t}≔\left[\begin{array}{c} \psi_{t}\\ {\dot{\psi }}_{t} \end{array}\right]=\left[\begin{array}{cc} I & 0\\ -{ad}_{\xi_{d,t}} & I \end{array}\right]x_{t}-\left[\begin{array}{c} 0\\ \xi_{d,t} \end{array}\right].$$ (10)

Given some semi-positive definite weights *P*, *Q*, and *R*, we can now write the quadratic cost function as

$$N\left(y_{t_{f}}\right)=y_{t_{f}}^{T}Py_{t_{f}},\;L\left(y_{t},u_{t}\right)=y_{t}^{T}Qy_{t}+u_{t}^{T}Ru_{t}.$$ (11)

Given the future twists *ξ*_*d*,*t*_, initial error state *ψ*_0_ and twist *ξ*_0_, we can define all the matrices. Discretizing the system at time steps ${\left\{t_{k}\right\}}_{k=1}^{N}$ , we can design the MPC as follows.

Problem 2. *Find* $u_{k}\in g^{*}$ *such that*

$$\begin{array}{ll} \underset{u_{k}}{\min}\; & y_{N}^{T}Py_{N}+\overset{N-1}{\underset{k=1}{\sum }}y_{k}^{T}Qy_{k}+u_{k}^{T}Ru_{k}\\ \text{s.t. } & x_{k+1}=A_{k}x_{k}+B_{k}u_{k}+h_{k},\;u_{k}\in U_{k},x_{0}=x\left(0\right). \end{array}$$

In Problem 2, *A*_*k*_, *B*_*k*_, and *h*_*k*_ can be obtained by zero-order hold or Euler first-order integration. Problem 2 is a QP problem that can be solved efficiently, e.g., using OSQP (Stellato et al., 2020).

### 4.3 Stability analysis

The stability of the proposed controller can be verified by a quadratic Lyapunov cost function in Lie algebra. First, we introduce the left invariant inner product. Then, we can derive the gradients of the quadratic cost function in the tangent space.

Definition 1. *Given* $\phi_{1},\phi_{2}\in R^{\dim g}$ *and* $\phi_{1}^{\wedge },\phi_{2}^{\wedge }\in g$ *, we define the inner product* ${\left\langle \phi_{1}^{\wedge },\phi_{2}^{\wedge }\right\rangle }_{g}=\phi_{1}^{T}P\phi_{2}$ *, where* *P* *is a positive definite matrix. This inner product is left-invariant. To see this, suppose* $X\phi_{1}^{\wedge },X\phi_{2}^{\wedge }\in T_{X}G$ *,* ∀X∈G *, then* ${\left\langle X\phi_{1}^{\wedge },X\phi_{2}^{\wedge }\right\rangle }_{X}={\left\langle {\left(\ell_{X^{-1}}\right)}_{*}X\phi_{1}^{\wedge },{\left(\ell_{X^{-1}}\right)}_{*}X\phi_{2}^{\wedge }\right\rangle }_{g}={\left\langle \phi_{1}^{\wedge },\phi_{2}^{\wedge }\right\rangle }_{g}$ *, where* ${\left(\ell_{X^{-1}}\right)}_{*}=X^{-1}:T_{X}G\to g$ *is the pushforward map.*

Theorem 1. *Consider the state* X∈G *,* $\phi \in R^{\dim g}$ *, and* *X* = exp(*ϕ*)*. We consider the metric in Definition* 1*. The function* $h=\frac{1}{2}\|\phi {\|}_{P}^{2}$ *is a candidate Lyapunov function and the gradient of* *h* *with respect to* *X* *is* ∇*h* = *Xϕ* ^∧^ *.*

Finally, we show that a linear feedback in Lie algebra can regulate the state to the identity exponentially.

Theorem 2. *Consider the state in Theorem* 1 *as a trajectory. Let* $\xi^{\wedge }\in g$ *. The system* $\dot{X}=X\xi^{\wedge }$ *can be exponentially stabilized to* *X* = *I* *by linear feedback* *ξ* = *Kϕ* *, where* *K* *is a gain matrix that is Hurwitz.*

The detailed proof of the theorems are presented in the work of Teng et al. (2022b). For the proposed MPC, we can follow the same steps and estimate the region of attraction. For the unconstrained case, the resulting LQR problem will lead to a linear feedback that can be verified by Theorem 2.

### 4.4 Validation on quadrupedal robot

We conduct two experiments on the quadrupedal robot Mini Cheetah (Katz et al., 2019) to evaluate the proposed MPC. Both experiments use a single rigid body model to approximate the torso motion. We apply MIT controller (Di Carlo et al., 2018) with the proposed MPC to plan the Ground Reaction Force (GRF).

#### 4.4.1 Robot pose tracking

**FIGURE 9:**
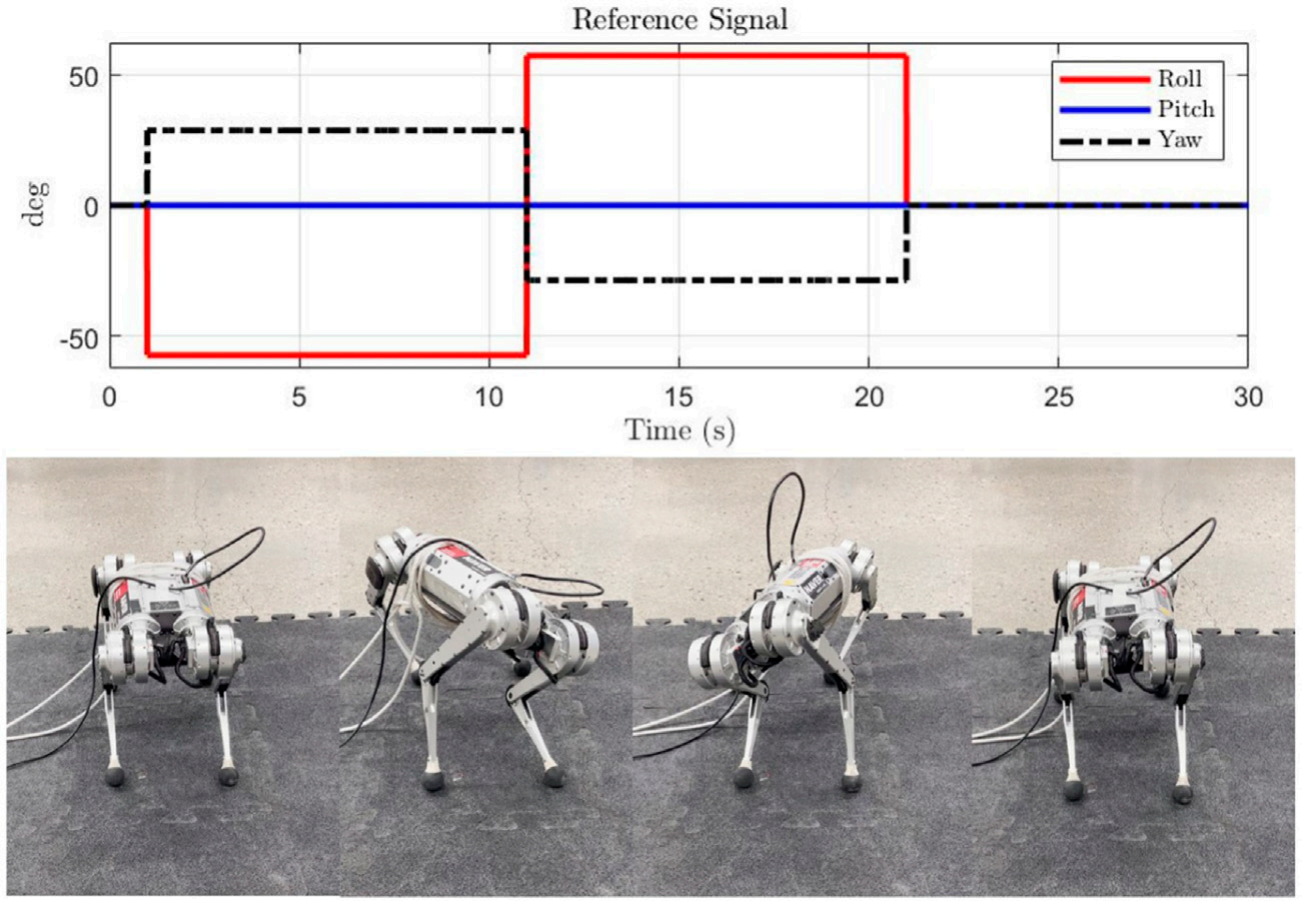
Reference signal for roll and yaw angle tracking. From 1 to 11 s, the robot roll changes from 0 to -57.3° and yaw changes from 0 to 28.5°. Then the robot leans to the opposite side for 10 s.

**FIGURE 10:**
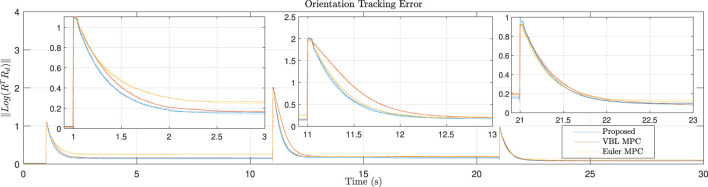
Error convergence for roll and yaw tracking. When a new step signal is applied, our controller converges faster than the baseline methods and has a smaller steady-state error. The Euler angle-based MPC has a larger steady-state error as both roll and yaw signals are applied.

In this experiment, a mixture of roll and yaw reference angle is applied for tracking. The reference signals and snapshots of robot motion are presented in Figure 9. Each controller is implemented three times. The details of the responses are presented in Figure 10. It can be seen that as no feedforward force at the equilibrium is provided, all controllers have steady-state error. However, the geometric-based controller, i.e., proposed and the VBL based MPC, has a smaller steady-state error than the Euler angle-based one. As the VBL based MPC does not conserve the scale of the error, the convergence rate is much lower than our controller, especially when the opposite Euler angle signal is applied at the middle of the reference profile.

#### 4.4.2 Robot trotting

We also apply our controller to robot locomotion. Ours and baseline controllers are deployed to plan the robot’s GRF given command twists. Then the GRF is applied to the Whole Body Impulse Control (WBIC) (Kim et al., 2019) to obtain the joint torques. Unlike the conventional whole-body controller, WBIC prioritizes the GRF generation by penalizing the deviation of GRF from the planned GRF. We increase the penalty for the GRF by 1e4 times in the original WBIC, so the GRF merely deviates from the planned one.

**FIGURE 11:**
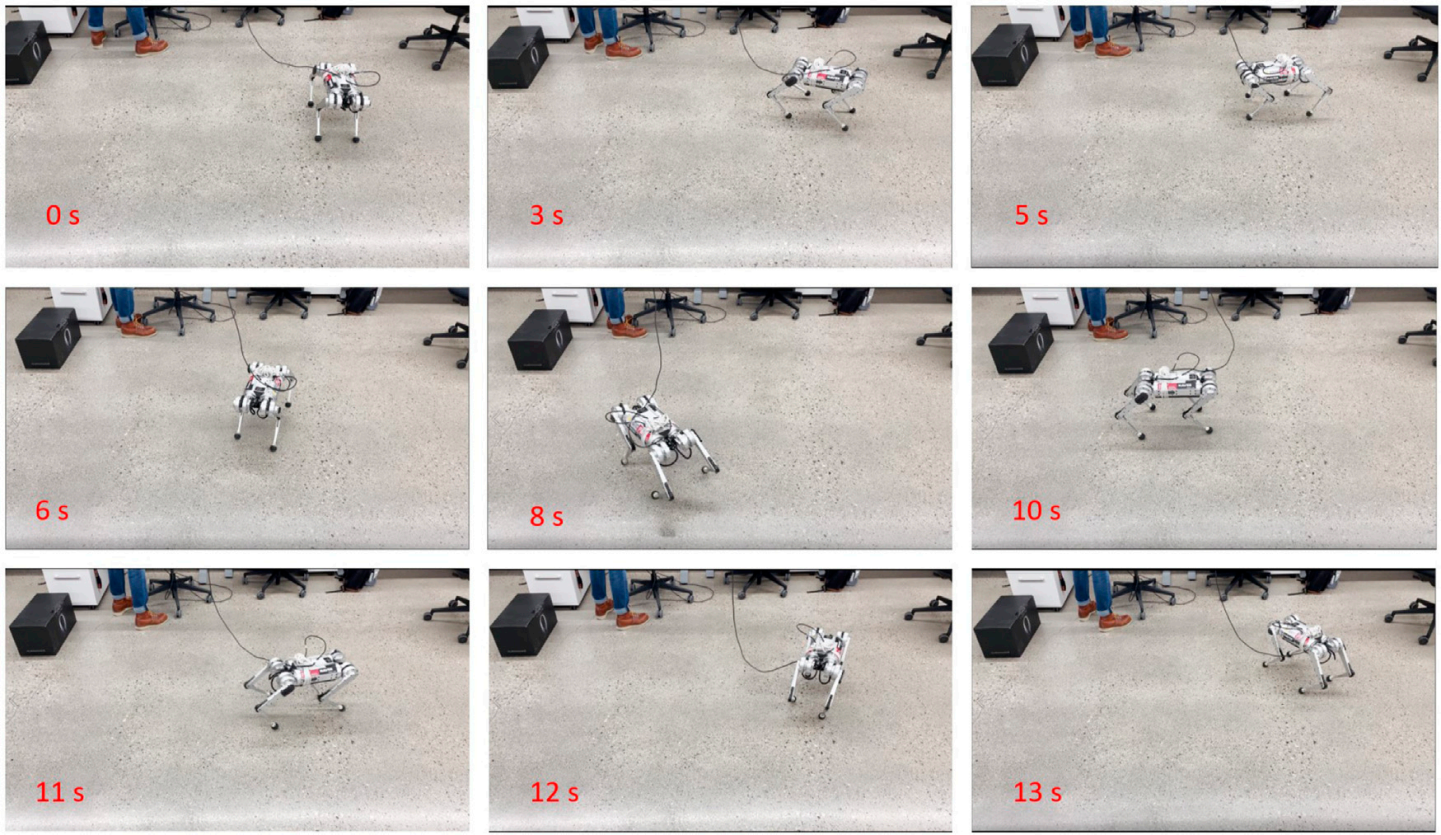
Snapshots of the experiments on reference tracking in Mini Cheetah trotting. The time corresponds to the reference signal in Figure 12.

**FIGURE 12:**
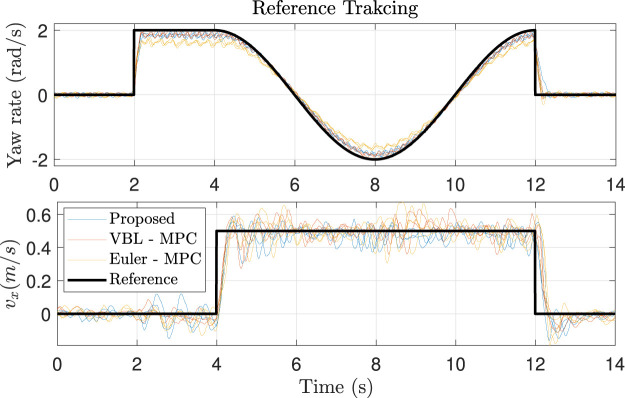
Reference tracking for quadrupedal robot trotting. Each controller is tested three times. The responses are too noisy; thus, the results are smoothed using the moving average filter.

We first apply a step signal in yaw rate. Then we add a step signal in *x* motion in the robot frame, and the yaw rate becomes a sinusoidal signal. The reference is presented in Figure 12 and the snapshots of the experiments are in Figure 11. We find that ours and the VBL-MPC can better track the yaw rate than the Euler angles-based MPC, as expected. As the orientation and position tracking errors are small because every step is integrated from the current state, it is reasonable that all controllers perform well in position tracking. The result can be seen in Figure 12.

## 5 Equivariant representation learning: Augmenting geometry with learning

Learning equivariant representation of geometric data can provide efficiency and generalizability in challenging robot perception tasks. Loosely speaking, *equivariance* is a property for a map such that given a transformation in the input, the output changes in a predictable way determined by the input transformation. Mathematically the equivariance is represented as commutativity: a function *f* : *X* → *X* is equivariant to a set of transformations *G*, if for any *g* ∈ *G*, *g* ⋅ *f*(*x*) = *f*(*g* ⋅ *x*), *∀x* ∈ *X*. For example, applying a translation on a 2D image and then going through a convolution layer is identical to processing the original image with a convolution layer and then shifting the output feature map. Therefore convolution layers are translation-equivariant.

An equivariant network captures the inherent symmetry of data, disentangling the information dependent on and independent of the transformations. As an analogy, this is akin to the notion of coordinates-free calculations on manifolds in modern mathematrics. In a coordinates-free setup, one can distinguish the intrinsic properties of the problem from those of a particular choice of coordinates (Tu, 2011). We mainly focus on the rigid body transformations, decoupling the poses and the pose-independent information, e.g., shapes and semantics, from the geometric data by leveraging equivariant feature learning.

### 5.1 Point cloud registration with SO(3)-equivariant implicit shape representations

**FIGURE 13:**
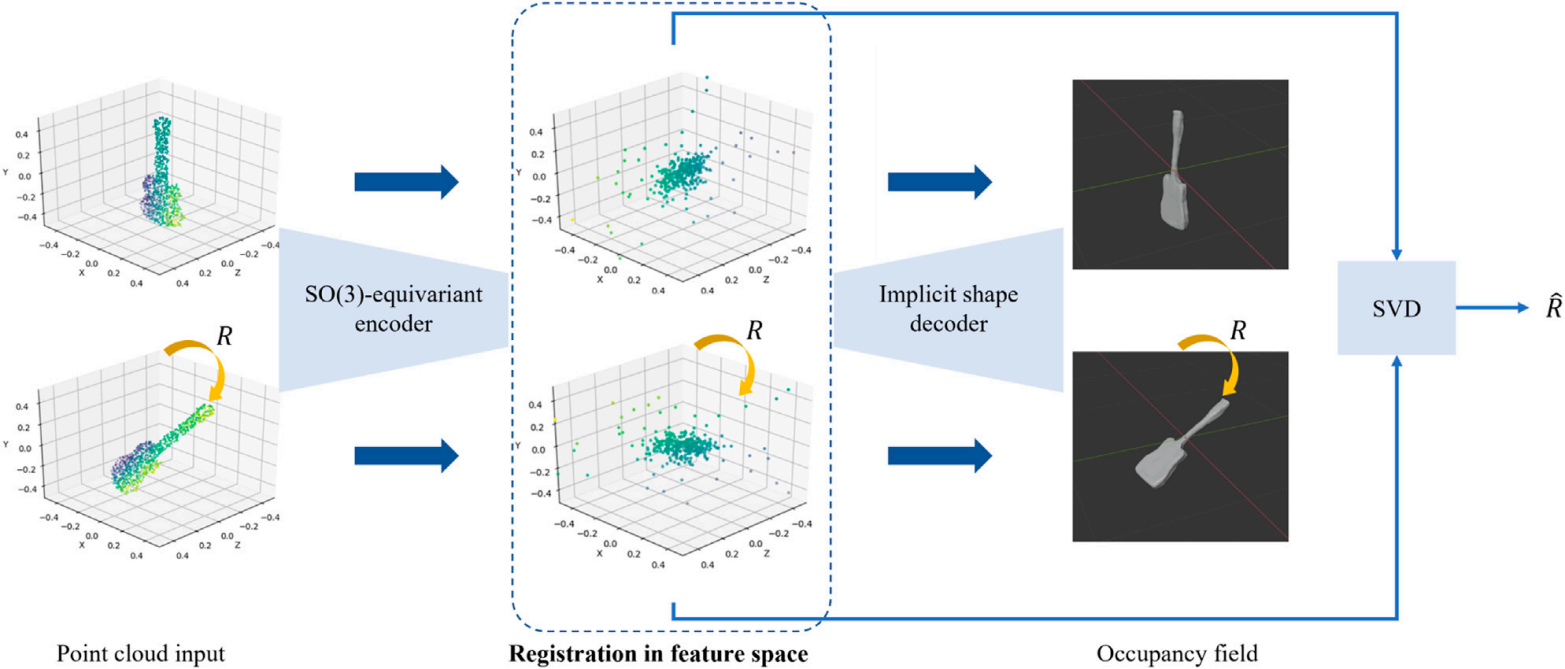
Overview of the SO(3)-equivariant registration network (Zhu et al., 2022b). The point cloud input is of shape $R^{N\times 3}$ , and the encoded feature is of shape $R^{C\times 3}$ . *N* is the number of points, and *C* is the dimension of features. Occupancy field is a function $v\left(p\right)\mapsto \left[0,1\right],p\in R^{3}$ mapping any 3D coordinate to an occupancy value. The rotation is estimated by aligning the features using Horn’s method (Horn et al., 1988).

We proposed an initialization-independent rotation registration method for point clouds by leveraging a SO(3)-equivariant feature learner (Zhu et al., 2022b). An overview of the network structure is depicted in Figure 13. A point cloud is mapped to a feature space equipped with SO(3) rotations represented as 3 × 3 matrix multiplications, consistent with the input Euclidean space. Therefore, the rotational registration can be approached by solving the Orthogonal Procrustes problem in the feature space. Our method achieved accurate rotation registration regardless of initial estimation error. It also implies that our method falls in the correspondence-free category, where the step of data association, i.e., matching corresponding points in two point clouds, is not needed.

The SO(3)-equivariant feature learning is realized through a backbone network called Vector Neuron (Deng et al., 2021). The key idea is to augment the scalar feature in each feature dimension to a vector in $R^{3}$ . In Vector Neuron networks, the feature matrix with feature dimension *C* corresponding to a set of *N* points is $V\in R^{N\times C\times 3}$ . The mapping between layers can be written as $f:R^{N\times C_{l}\times 3}\to R^{N\times C_{l+1}\times 3}$ , where *l* is the layer index. Following this design, the representation of SO(3) rotations in feature space is straightforward: *g*(*R*) ⋅ *V*≔*VR*, where *g*(*R*) denotes the rotation operation in the feature space, parameterized by the 3 × 3 rotation matrix *R* ∈ SO(3). Here we ignore the first dimension *N* of *V* for simplicity, and the SO(3)-equivariance of the linear layer: *f*_lin_(*V*) = *WV*, where $W\in \;R^{C_{l+1}\times C_{l}}$ , can be easily verified as follows.

$$g\left(R\right)\cdot f_{\text{lin}}\left(V\right)=WVR=f_{\text{lin}}\left(g\left(R\right)\cdot V\right).$$ (12)

For further discussions beyond the linear layers, see the work of Deng et al. (2021).

We design an encoder-decoder structure to learn the features. We also improve the robustness to noise in sampled points by decoding an implicit shape representation following the Occupancy network (Mescheder et al., 2019). Our method is tested on the synthetic object-wise data set ModelNet40 (Wu et al., 2015), shown in Table 5. For further experiments using real-world indoor RGB-D data set 7Scenes (Shotton et al., 2013), see the work of (Zhu et al., 2022b).

**TABLE 5:** Rotational registration error given rotated copies of point clouds. Tested using ModelNet40 (Wu et al., 2015) official test set. The best are shown in **bold**. The second best are shown in *italic*. All values are in degrees.

Max initial rotation angle			0	30	60	90	120	150	180
Categories	Global	Methods	Rotation error after registration						
Correspondence-free	N	PCR-Net Sarode et al. (2019)	7.08	9.50	27.38	68.22	109.61	129.29	133.49
	N	FMR Huang et al. (2020)	**0.00**	0.45	5.29	21.95	42.26	66.39	79.43
	Y	Ours Zhu et al. (2022b)	*0.02*	**0.02**	**0.02**	**0.02**	**0.02**	**0.02**	**0.02**
Correspondence-based	N	RPM-Net Yew and Lee, (2020)	0.26	0.27	0.42	1.57	2.85	3.42	4.02
	Y	DeepGMR Yuan et al. (2020)	*0.02*	**0.02**	**0.02**	**0.02**	**0.02**	**0.02**	**0.02**

Bold values in tables show the best performance.

### 5.2 Efficient SE(3)-equivariant representations learning

Our recent work (Zhu et al., 2022a) extends the SO(3)-equivariance to SE(3)-equivariance to better deal with arbitrary rigid body transformations of 3D point-cloud data. We use Convolutional Neural Networks (CNNs) which inherit translational equivariance. Existing work of equivariant convolutional networks are mainly in two types. First is regular G-CNNs (G for *group*) (Cohen and Welling, 2016), which lift the domain of the feature function space from the input Euclidean space to the group of transformations of interest. Second is steerable G-CNNs (Thomas et al., 2018), which leave the domain of the feature function space untouched but design the codomain to be *steerable* with the stabilizer subgroup. More detailed introductions can be found in the work of Cohen et al. (2018). The former strategy consumes much larger memory than a conventional CNN, while the latter usually results in complex design and restrictions on the kernel and convolution structure, both limiting broader applications in practice. We propose a new strategy to lift the domain of feature space to a proper subgroup of SE(3), and to apply a trivial steering representation on the subgroup, which addresses both problems mentioned above. Our proposed point-cloud convolution network learns expressive SE(3)-equivariant features with a much smaller footprint than existing methods. See Table 6 for a comparison between our method and a baseline regular G-CNN method, EPN (Chen et al., 2021).

**TABLE 6:** Experiment result of pose estimation on ModelNet40 data set (Wu et al., 2015) on the plane category. Two numbers are shown for GPU memory consumption and running speed for training/inference separately, given the same input size for two methods. Notice that the numbers are not directly comparable to Table 5 due to different experiment settings.

Methods	Memory (GB) *↓*	Speed (fps) *↑*	Mean error (°) *↓*	Median error (°) *↓*	Max error (°) *↓*
EPN Chen et al. (2021)	22.2/16.9	1.1/1.6	1.25	1.11	6.63
*Ours* Zhu et al. (2022a)	**4.3**/**2.8**	**6.7**/**11.1**	**1.17**	**1.08**	**5.90**

Bold values in tables show the best performance.

**FIGURE 14:**
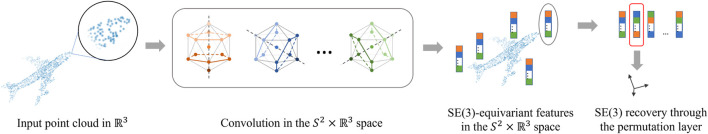
A high-level illustration of our efficient SE(3)-equivariant network. We lift the convolution to $S^{2}\times R^{3}$ , which is a rare choice for SE(3)-equivariant feature learning. The different colors represent channels in *S* ^2^.

To be more specific, our convolution structure is built upon KPConv (Thomas et al., 2019). We choose SO(2) as the stabilizer and work with feature maps defined on the domain $\tilde{X}=\text{SE}\left(3\right)/SO\left(2\right)$ which is homeomorphic to the Cartesian product $S^{2}\times R^{3}$ . We extend the KPConv from $R^{3}$ to $S^{2}\times R^{3}$ . We discretize SO(3) into the icosahedral rotation group I with 60 elements, following EPN by Chen et al. (2021), containing all rotational symmetries of an icosahedron. SO(2) is discretized as the group of multiples of 72° planar rotations, which is a cyclic group of degree 5. Then we obtain a discretization of the sphere $\bar{S^{2}}=\bar{SO\left(3\right)}/\bar{SO\left(2\right)}$ of size 12 corresponding to the vertices of an icosahedron, where $\bar{\cdot }$ (a top bar) denotes the discretized space. As a result, the domain of feature maps in our network is $\bar{S^{2}}\times R^{3}$ . It turns out that we can design an SE(3)-equivariant convolution in this space in a simple and efficient form while maintaining expressiveness. The full $\bar{SO\left(3\right)}$ information can be recovered from the $\bar{S^{2}}$ feature maps through a permutation layer. An overview of the network structure is shown in Figure 14.

### 5.3 Place recognition via SE(3)-invariant representation

Place recognition, also known as loop closure detection, enables a robot to determine if it has seen a place before and provides loop closure candidates for SLAM algorithms to eliminate accumulated error. The widely used sensors include RGB, Stereo, Thermal, Event-Triggered, and RGB-D, which are in the form of 2D structured images or 3D unstructured points (Barros et al., 2021). For general tasks with 2D images, place recognition tasks suffer less because the training and testing images differ trivially in roll direction during data collecting procedures. Yet, the roll angles deviate significantly in challenging scenarios like surgery (Song et al., 2021), underwater robot (Li et al., 2015) or special camera setup in general cases. Orientational differences widely exist and pose great difficulty to place recognition with 3D unstructured point cloud perception. Therefore, place recognition methods can benefit from a representation that is robust to arbitrary transformations of 3D point cloud data.

The image-based localization can be categorized as constructing hand-crafted rotation-invariant descriptors in 2D (Cummins and Newman, 2008; Gálvez-López and Tardos, 2012), learning the global descriptor (Kendall et al., 2015; Sünderhauf et al., 2015; Kim et al., 2017) or a combination of both (Tian et al., 2020; Song et al., 2022). Although learning-based methods achieve better accuracy and robustness, Lowry et al. (2015) suggested that place-recognition scenarios with large orientation differences still rely on hand-crafted descriptors which are designed for robust feature matching. This is especially true for 3D point clouds suffering more from orientation differences. Existing point cloud-based place recognition methods improve the transformation robustness by extracting 3D hand-crafted rotation-invariant descriptors (Kim and Kim, 2018; Wang et al., 2019; Yin et al., 2019; Kim et al., 2021) and randomly rotating them during training (Uy and Lee, 2018; Cattaneo et al., 2021). However, hand-crafted features can lose structural information and these methods do not take translation into consideration.

**FIGURE 15:**
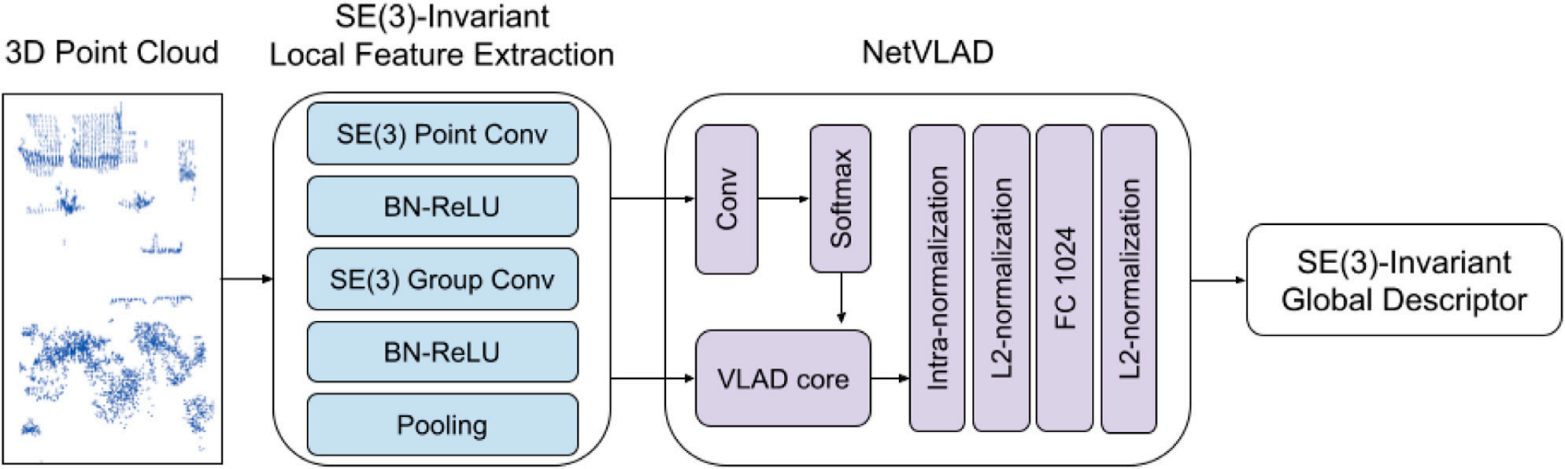
Overview of the SE(3)-invariant place recognition network. In this network, SE(3)-invariant features are learned from input point clouds. The local feature extraction consist of SE(3) point convolution, SE(3) group convolution, batch normalization followed by leaky ReLU activation, and pooling layer which makes equivariant features invariant. Global descriptors are computed by aggregating local features using NetVLAD. The output descriptors can perform place recognition tasks.

To avoid an exhaustive data augmentation with all possible transformations and improve generalizability, we propose an SE(3)-invariant place recognition representation network for the 3D point cloud. An overview of the network structure is shown in Figure 15. We use EPN (Chen et al., 2021) to extract SE(3)-invariant local features. NetVLAD (Arandjelovic et al., 2016) is applied to aggregate local features and construct SE(3)-invariant global descriptors.

**FIGURE 16:**
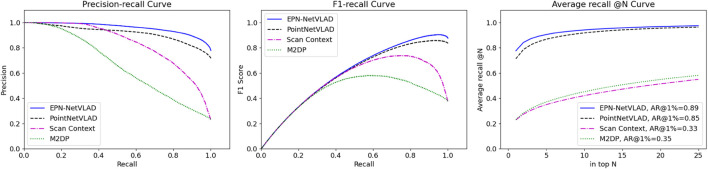
Experimental results of proposed method (EPN-NetVLAD, blue line), state-of-the-art approaches PointNetVLAD (Uy and Lee, 2018), Scan Context (Kim and Kim, 2018), and M2DP (He et al., 2016) on Oxford RobotCar benchmark.

We evaluate the proposed place representation using the Oxford RobotCar (Maddern et al., 2017) benchmark created by Uy and Lee (2018). The precision-recall curves of the proposed method and other baseline methods are shown in Figure 16. The proposed network EPN-NetVLAD outperforms the baselines. To show the generlizability of the proposed method, we experiment with three in-house data sets of a university sector (U.S.), a residential area (R.A.) and a business district (B.D.) (Uy and Lee, 2018). The result is shown in Table 7 and our method performs better in all the data sets that we did not train on.

**TABLE 7:** Experiment result showing the average recall (%) at top 1% for each of the models. Both methods are only trained on Oxford (Maddern et al., 2017) and tested on other different data sets (Uy and Lee, 2018).

Datasets	Oxford	U.S.	R.A.	B.D.
PointNetVLAD Uy and Lee, (2018)	84.94%	80.79%	73.86%	69.29%
EPN-NetVLAD (Ours)	**89.17%**	**87.82%**	**81.98%**	**76.91%**

Bold values in tables show the best performance.

## 6 Closing remarks and future opportunities

Autonomy via computational intelligence is a multifaceted research domain that nicely integrates mathematics, computer science, and engineering and can have enormous impacts on our future and improve our quality of life. Robotics plays a unique role by connecting the real world to AI, i.e., embodied AI. Many challenges in robotics are natural problems in AI because they show what it takes to develop an autonomous system capable of operating in the wild. We reviewed some of the recent efforts in symmetry-preserving robot perception and control methods. In particular, by symmetry, we refer to invariance or equivariance properties under a group action enabled by Lie groups or their discrete subgroups.

The RKHS registration framework presented in Section 2 provides a unified model for registration that jointly integrates geometric and semantic measurements and does not require explicit data association. This framework is intimately connected with deep learning models. The inner product of the functions viewed as cross-correlation can be modeled as a network layer to combine the power of functional modeling with feature and kernel learning. Moreover, since the framework is equivariant, it can be directly combined with equivariant feature learners, e.g., via deep kernel learning (Wilson et al., 2016). An important open problem is a relationship among our framework, discrete-continuous smoothing and mapping (Doherty et al., 2022), dynamic scene graphs (Rosinol et al., 2021), and learning-aided smoothing and mapping (Huang et al., 2021) for robot perception and navigation. These are attractive research directions that we will explore in the future.

The learning-aided state estimation framework, presented in Section 3, can be extended to multi-task networks (Liu et al., 2019; Maninis et al., 2019; Hu and Singh, 2021) for tasks such as slip detection and friction coefficient estimation (Focchi et al., 2018; Romeo and Zollo, 2020), terrain classification (Hoepflinger et al., 2010; Walas et al., 2016; Wu et al., 2016; Ahmadi et al., 2021), covariance estimation (Brossard et al., 2020), sensor calibration and integration (Liu et al., 2020; Brossard et al., 2022; Ji et al., 2022), and motion mode detection (Brossard et al., 2019). A high-frequency implementation of these works on robots can significantly improve their capabilities for navigating challenging environments. Moreover, the work of Hwangbo et al. (2019) designs a learning-based controller using a policy network that maps kinematic observations and the joint state history to the joint position targets. Then an actuator network takes the joint velocity history and joint position error history to learn the joint torque. The success of Hwangbo et al. (2019) suggests that our multimodal approach to learning can improve the controller performance while further optimization of the contact estimation network size is possible.

In Section 4, we developed a new error-state MPC approach on connected matrix Lie groups for robot control. By exploiting the existing symmetry of the pose control problem on Lie groups, we showed that the linearized tracking error dynamics and equations of motion in the Lie algebra are globally valid and evolve independently of the system trajectory. In addition, we formulated a convex MPC program for solving the problem efficiently using QP solvers. A Lyapunov function expressed in Lie algebra is introduced to verify the exponential stability of the proposed controller. The experimental results confirm that the proposed approach provides faster convergence when rotation and position are controlled simultaneously. Future work will implement the trajectory optimization using this geometric control framework proposed by Teng et al. (2022b) for robot control. Another interesting research direction is to incorporate learning into this framework (Shi et al., 2019; Li et al., 2022; Ma et al., 2022; O’Connell et al., 2022; Power and Berenson, 2022; Rodriguez et al., 2022). In addition, the IIG algorithm (Ghaffari Jadidi et al., 2019), combined with an MPC (Teng et al., 2021a), can provide an integrated kinodynamic planner that takes the robot stability, control constraints, and the value of information from sensory data into account. Gan et al. (2022) show that the value of information can be learned from multimodal sensory input via learning from demonstrations and self-supervised trajectory ranking to deal with sub-optimal demonstrations.

In Section 5, we showed how equivariant neural networks can serve as powerful feature learners to improve data efficiency and generalizability across different tasks. In particular, we provided results on registration and place recognition tasks. We argue that our efficient SE(3)-equivariant network (Zhu et al., 2022a) can be a reliable feature learner for a variety of robot perception and control problems, including those mentioned in this article. Furthermore, this symmetry-preserving representation can be an answer to the long-standing question of a “good” representation for robot mapping.

In addition to the point cloud-based SE(3)-invariant place recognition, it is of great interest to investigate the image-based version in challenging scenarios ranging from unstructured outdoors to endoscopy and colonoscopy (Song et al., 2021). Cohen and Welling (2016), Cohen et al. (2018) provide valuable insights on equipping the existing learning-based algorithms with group-invariant feature extraction ability. Traditional hand-crafted descriptors can be substituted with learnable deep SE(3)-invariant image descriptors. More importantly, we believe a natural future direction for robotics is towards developing structure-preserving and correct-by-construction computational models, such as our SE(3)-equivariant network, to enable efficient and generalizable multimodal learning.

Finally, this article aims to serve as an invitation to developing algorithms that respect the geometry of problems in robotics, preserve structures such as symmetry, and use modern computation methods such as deep learning. We presented methods ranging from purely geometric to end-to-end learning. As such, the central message of this paper is not about outperforming a particular framework, but it lies in the combined power of geometry and learning and the possibility of modeling traditional geometric problems using geometric networks such as equivariant deep networks. The latter will lead to explainable large-scale computational models for robotics and autonomous systems.

## Data Availability

The datasets presented in this study can be found in online repositories. The names of the repository/repositories and accession number(s) can be found in the article/supplementary material.

## Footnotes

1
https://github.com/UMich-CURLY/deep-contact-estimator; https://github.com/UMich-CURLY/cheetah_inekf_realtime; https://github.com/UMich-CURLY/husky_inekf.
